# Supplementary material for: Psychosocial interventions for adults with visible differences: a systematic review
Source: PeerJ. 2015 Apr 2;3:e870. doi: 10.7717/peerj.870 (PMC4389275; doi:10.7717/peerj.870)
Supplement: Appendix — A: sample search strategy [file peerj-03-870-s001.docx]

**Appendix A: Sample search strategy**

1 exp Adaptation, Psychological/

2 exp Psychotherapy/

3 exp Counseling/

4 "Self-Help Groups"/

5 "Social Support"/

6 ((psychosocial$ or psycho-social$) adj5 (intervention$ or treatment$ or therap$ or program$)).tw.

7 counsel$.tw.

8 (behavi$ adj5 (therap$ or treatment$ or program$ or intervention$)).tw.

9 (cognitiv$ adj5 (therap$ or treatment$ or program$ or intervention$)).tw.

10 (psychologic$ adj5 (therap$ or treatment$ or program$ or intervention$)).tw.

11 (mindfulness adj5 (therap$ or treatment$ or program$ or intervention$)).tw.

12 "Early Intervention (Education)"/

13 Patient Education as Topic/

14 support group$.tw.

15 self-help.tw.

16 psychotherap$.tw.

17 group therap$.tw.

18 Social Adjustment/

19 person-cent$ therap$.tw.

20 solution-based therap$.tw.

21 or/1-20

22 exp Cicatrix/

23 ((face or facial) adj3 scar$).tw.

24 (visible adj3 scar$).tw.

25 keloid$.tw.

26 cicatrix.tw.

27 exp Facial Injuries/

28 ((facial$ or face) adj3 (injur$ or damage$)).tw.

29 exp Craniofacial Abnormalities/

30 exp Facial Dermatoses/

31 facial dermatos$.tw.

32 Psoriasis/

33 psoriasis.tw.

34 Eczema/

35 eczema.tw.

36 exp Skin Abnormalities/

37 Epidermolysis Bullosa.tw.

38 port wine stain$.tw.

39 exp Hemangioma/

40 h?emangioma$.tw.

41 exp Pigmentation Disorders/

42 vitiligo.tw.

43 exp "nevi and melanomas"/

44 (birth mark$ or birthmark$).tw.

45 melanoma$.tw.

46 burns/

47 burns.ti.

48 exp Alopecia/

49 alopecia.tw.

50 exp Exophthalmos/

51 exophthalm$.tw.

52 thyroid eye disease.tw.

53 exp Strabismus/

54 strabismus.tw.

55 (misalign$ adj3 eye$).tw.

56 exp Eyelid Diseases/

57 exp Mouth Neoplasms/

58 ((mouth or oral) adj3 (neoplasm$ or cancer$ or tumo?r$)).tw.

59 Growth Disorders/

60 exp Dwarfism/

61 Gigantism/

62 dwarfism.tw.

63 dwarf.tw.

64 small stature.tw.

65 gigantism.tw.

66 restricted growth.tw.

67 exp Dystonia/

68 Torticollis.tw.

69 dystonia.tw.

70 Dupuytren Contracture/

71 Dupuytren$ contracture$.tw.

72 Amputation/

73 Artificial Limbs/

74 Amputees/

75 amputee$.tw.

76 artificial limb$.tw.

77 (appearance adj5 (abnormal or malformation or problem$)).tw.

78 (visibl$ adj5 disabilit$).tw.

79 (visibl$ adj5 differen$).tw.

80 disfigur$.tw.

81 (appearance adj5 (malform$ or problem$)).tw.

82 (deformit$ or deformed).tw.

83 (appearance$ adj5 (distress or anxiety or depression)).tw.

84 appearance.ti.

85 exp Mouth Abnormalities/

86 hare lip$.tw.

87 harelip$.tw.

88 Palatoschisis.tw.

89 cleft lip$.tw.

90 cleft palate$.tw.

91 orofacial$ cleft$.tw.

92 facial cleft$.tw.

93 oral cleft$.tw.

94 craniofacial cleft$.tw.

95 or/22-94

96 21 and 95

97 exp animals/ not humans/

98 96 not 97

99 limit 98 to "all adult (19 plus years)"

100 limit 98 to "all child (0 to 18 years)"

101 100 not 99

102 98 not 101
